# Providing Excellent Customer Service Is Therapeutic: Insights from an Implicit Association Neuromarketing Study

**DOI:** 10.3390/bs9100109

**Published:** 2019-10-14

**Authors:** Gemma Anne Calvert, Abhishek Pathak, Lim Elison Ai Ching, Geraldine Trufil, Eamon Philip Fulcher

**Affiliations:** 1 Nanyang Business School, Nanyang Technological University, 50 Nanyang Avenue, Singapore 639798, Singapore; LIMAC@ntu.edu.sg; 2 School of Business, 4 Nethergate, University of Dundee, Dundee DD1 4HN, UK; a.z.pathak@dundee.ac.uk; 3 Split Second Research Limited, London E1 8FA, UK; geraldine.trufil@splitsecondresearch.co.uk (G.T.); eamon.fulcher@splitsecondresearch.co.uk (E.P.F.)

**Keywords:** customer service, employee retention, semantic priming, implicit reaction time, cognitive neuroscience, neuromarketing

## Abstract

This paper reports the results of a combined biometric and implicit affective priming study of the emotional consequences of being the provider or receiver of either positive or negative customer service experiences. The study was conducted in two stages. Study 1 captured the moment-by-moment implicit emotional and physiological responses associated with receiving and providing good customer service. Study 2 employed an affective priming task to evaluate the implicit associations with good and poor customer service in a large sample of 1200 respondents across three Western countries. Our results show that both giving and receiving good customer service was perceived as pleasurable (Study 1) and at the same time, was implicitly associated with positive feelings (Study 2). The authors discuss the implications of the research for service providers in terms of the impact of these interactions on employee wellbeing, staff retention rates and customer satisfaction.

## 1. Introduction

Customer satisfaction is a vital goal for all businesses because it leads to increased sales and customer re-patronage, which ultimately boosts profits. To this end, managing customer experiences across the customer–employee touchpoints plays a critical role, given that most businesses involve some level of direct contact (e.g., face-to-face or voice-to-voice) between employees (especially those working at the consumer interface) and customers. Yet delivering high quality and effective customer service is not a straightforward or easily managed process. Customer–employee interactions have a significant emotional component that often confounds training strategies. While it is understood that positive customer service results in better marketing outcomes, much less is known about the emotional impact on those responsible for delivering that service.

Service employees often hide their true inner feelings and maintain a pleasant facial and bodily display in a bid to please their customers and/or gain control over employee–customer interactions [1,2]. Indeed, companies often train their service employees to act in a friendly manner since the display of positive emotions is associated with favourable consequences, such as increased customer satisfaction, customer re-patronage, and positive word-of-mouth [3]. Such acting requires significant effort on the part of the employees and can cause employees to suffer emotional burn-out if they are required to “put on” displayed emotions for long periods of time [4,5]. Furthermore, consumers do not always appreciate employee friendliness, and may even construe it negatively as being disrespectful [6]. Indeed, consumers are increasingly adept at discerning the expressive behaviour of service providers. For instance, they are more likely to be moved by the authenticity of an employee’s smile rather than the extent of it [7,8]. The somewhat artificial nature of these exchanges, coupled with the constant requirement to suppress negative emotions and “appear” friendly and understanding, makes it extremely difficult to disentangle true emotions associated with positive and negative customer–staff interactions and those which individuals presume they should experience.

Measuring the emotional consequences associated with customer experience is further complicated by the fact that it involves multiple moments of contact between an organisation and a customer. These may include the feelings evoked when walking into a shop, the way in which the customer is treated by frontline service employees in-store, as well as post-purchase follow-up customer service. Furthermore, the ability of individuals to introspect and comment on the nature of these subjective emotional responses, particularly during dynamic social interactions, is highly variable and often inaccurate [9,10]. The relationship (and perceptions thereof) between the employee-customer interaction has traditionally been measured using surveys [11] (may not be accurate always, and we propose an alternate method in this the current paper.

Extant scholarly researchers as well as companies interested in assessing service quality mostly employ explicit, self-reported measures. However, this approach captures only a partial picture of the multitude of responses in consumers’ brains. Neuroscience research has shown that a vast amount of human behaviour is driven and influenced by emotional and cognitive responses that occur below conscious awareness [12,13,14]. At the conscious level, customers tend to know what they want and also how they wish to be treated. But important implications of good and poor customer service can also play out at the subconscious or implicit level of cognition [15,16]. The same is true for those responsible for providing that service, where multiple conscious and subconscious emotional factors impinge on the effectiveness of customer interactions. 

Although it is well known that the quality of customer–employee interaction is crucial for organisations and the importance of customer service has been studied for many years now, the literature is scarce on the consequences of poor (or good) service on employees [17,18]. Poor customer–employee interaction can lead to employee stress and is a potential health risk [19], which can cost up to $ 300 billion in losses cumulatively to organisations the world over American Institute of Stress (2014). Employees who are regularly tasked to maintain positive interactions with customers have also been reported to show excessive emotional burden, exhaustion and absenteeism [20,21]. Similarly, customer mistreatment (and consequent stress) can compromise both short term and long-term employee well-being [22] and result in emotional exhaustion [23]. 

Recent research has also shown that positive customer behaviour during service interactions has a cross over positive effect on the employee [24]. Similarly, stressful customer interactions can have a negative impact on the affective state of employees [25].

In order to develop a deeper understanding of the implicit consequences of customer service on providers and receivers, this research examined the implicit emotional responses associated with receiving and providing excellent service. Specifically, the paper investigated 1) the perception of both giving and receiving good vs. bad customer service, 2) and the implicit associations (or feelings) which people associate with the experience of giving or receiving good vs. bad customer service. By doing so, this research contributes to the services literature by demonstrating how the positive benefits of excellent customer service can impact not only on customers, but also on service providers themselves. Such positive outcomes, if made explicit, can clearly be exploited in a positive way so as to increase employee job satisfaction and reduce staff turnover rates. Furthermore, this research also contributes to the field by proposing a new research approach that captures customers’ *subconscious* responses in order to gain a more comprehensive understanding of the subliminal effects of positive customer–employee interactions.

## 2. Background

Over the past decade, techniques that have emerged from the fields of neuroscience and psychology, such as functional MRI, electroencephalography (EEG), eye-tracking, biometrics, facial decoding and implicit association testing, have been engaged by brand owners to capture these vital subconscious responses in order to define and predict consumer behaviour with much greater accuracy (for a recent review, see [9]). This approach has been referred to as “neuromarketing” [26] and numerous commercial practitioners of this burgeoning industry now exist. In recent years, commercial entities have paid particular attention to neuromarketing methods that are scalable, cost-effective and offer fast turnaround times [27]. 

One methodology that satisfies these criteria is the use of implicit reaction time tests [28]. The mainstay of many cognitive psychology experiments since the 1970s, implicit reaction time paradigms measure individuals spontaneous or ‘gut instinct’ responses. Commercial adaptations of these paradigms permit marketers to capture these vital subconscious consumer responses online, without the need for verbal feedback or even respondents’ awareness of their reactions. Implicit measures have now been used in a variety of settings to extract people’s implicit emotions and attitudes to a wide range of different issues, including racial prejudice, sexual preferences, alcoholism, mental health, and consumer attitudes (see [29] for an overview). Importantly, the implicit responses obtained in these studies were shown to be more predictive of respondents’ subsequent behaviour than their explicit verbal responses obtained at the same time and are therefore, in many instances, more accurate indicators of their emotional responses to specific concepts and scenarios.

Several recent implicit reaction time paradigms have been shown to have high reliability and validity [30,31,32,33]. These approaches rely on a simple behavioural response—a very rapid key press to the presentation of a stimulus, which is made following a simple decision about the stimulus. There are several distinct implicit paradigms, each with specific strengths and weaknesses, and the choice of task depends on the research question being addressed [29].

In the current study, we employed two implicit reaction time tests. The first was the Impulse Test recently developed and shown to measure the emotions evoked as respondents view *dynamic* material (e.g., while watching a television advertisement, movie trailer or video footage [34]. The second was an affective semantic priming task [35,36] that assesses the strength of implicit association between a set of emotional words and specific concepts, in this case, good and poor customer service. The rationale for employing two distinct implicit tests was that in the first case, we were able to identify the immediate emotions elicited by positive customer service interactions (both from the perspective of the provider and the receiver) and relevant in short-term memory, and in the second case, we were able to capture the more deep-seated emotions associated with positive as well as negative customer interactions that are stored in long-term memory.

Physiological responses (heart and breathing rate and electrodermal changes) were also measured during the Impulse test to determine if positive customer service interactions (both providing and receiving) impact the levels of arousal. Arousal, one of the components of emotional responding, is associated with stress, anxiety and fear [37], and physiological manifestations of arousal include increased blood pressure, heart rate, sweating and hyperventilation [38]. We hypothesized that the act of simply observing positive customer–staff interactions would result in reduced arousal and therefore stress levels, similarly to that experienced when engaging in other everyday pleasures.

This study was conducted in two stages. Study 1 was designed with two objectives in mind: first, the study served to identify the nature of the immediate emotions elicited in real time as respondents viewed videos of people receiving or providing excellent customer service compared with viewing other positive scenarios (e.g., everyday pleasurable activities such as enjoying time with friends), and secondly, we wanted to examine the physiological responses (heart and breathing rate, and electrodermal response) to the customer service scenarios depicted in the videos. In Study 2, we examined the more deeply held emotions (i.e., those maintained in long term memory) associated with customer service interactions (positive and negative) in a larger population (N = 1200) across three countries that individuals have either delivered or received.

## 3. Methods

### 3.1. Study 1: Laboratory Based Study

#### 3.1.1. Participants

Twenty participants (thirteen females (two left-handed) and seven males (all right-handed) with mean age of 27 years) were recruited from Bristol, UK (via flyers in exchange for vouchers) and given small incentives to take part in a study to measure their immediate physiological and psychological responses to different emotional scenarios in real-time, including footage depicting individuals providing or receiving customer service (sample size is similar to other studies of comparable nature, e.g., [39]).

#### 3.1.2. Materials

Three distinct video clips, each one minute in duration, were professionally created specifically for this study: 

*Video 1* (Condition 1: Control): was made up of footage of everyday pleasures (unrelated to customer service) shown from the first person perspective, such as eating crisps, going for a walk in the park.

*Video 2* (Condition 2: Providing excellent customer service): constituted footage of four different scenarios in which service staff were filmed delivering excellent customer service and the footage shown from the service provider’s perspective. The scenarios were as follows: (i) a booking agent is seen giving a customer tickets to a previously sold out play at the theatre and knowing she has had a hard time recently, the booking agent has gone one step further and arranged for her to go to the opening night party as well, (ii) a travel agent helps a couple, who have been separated for six months due to work, to plan their dream honeymoon, giving them personalised recommendations on where to visit and restaurants to eat out at, (iii) a groom leaves his wedding rings in the back of a taxi the day before the wedding. The taxi driver returns to the hotel where he dropped off the groom off, re-uniting him with the rings and thus saving the day, and (iv) a woman collapses in a restaurant while on holiday after which a fellow customer, a doctor, tries to help but her friend is very distressed and does not speak the local language. The waitress steps in to translate what the doctor is saying and accompanies them all to hospital.

*Video 3*: (Condition 3: Receiving excellent customer service): shows scenarios featuring excellent customer service and are the same scenarios as those used in Condition 2 but re-filmed and shown from the customer’s perspective.

#### 3.1.3. Protocol

Only one subject at a time participated in the experiment. Each participant was greeted by the experimenter who explained that the purpose of the study was to gain a better understanding of customer service interactions. After obtaining informed consent (FREC-EF02-PSY-16-1-2013), physiological electrodes were applied and subjects were seated in front of a computer screen. Heart and breathing rate as well as skin conductance measures were collected as subjects viewed the video clips. The experimental videos were shown on a computer screen and participants’ responses were recorded using the computer keyboard. The order of presentation of the three videos was counterbalanced across subjects.

BIOPAC physiological equipment was used in the collection of data. In order to measure heart rate, one electrode was placed on the medial surface of each leg just above the ankle. A third electrode was placed on the right anterior forearm at the wrist. Once electrodes were attached, participants were asked to remain still while the system parameters were calibrated. Data was recorded at a rate of 200 times a second. Heart rate was measured as the milliseconds between heart beats and was analyzed as the average heart-rate per two seconds (400 datapoints).

Skin conductance data was collected through two electrodes attached to the middle and ring finger of the non-dominant hand. The index finger was avoided as it was used in the reaction time task. Participants were given the opportunity to practice key pressing with minimal movement of the hand, so as not to disturb recording. Respiratory cycle was recorded through a respiratory transducer attached around the chest below the armpits and over the shirt. It was adjusted so that it was slightly tight at the point of maximal expiration.

During the acquisition of biometric data, subjects were also asked to carry out an implicit reaction time test (the Impulse test) while viewing the experimental videos. The Impulse test consists of two stages—a baseline phase and an experimental phase. In the baseline phase of the current task, participants were exposed to a set of emotional words (see Table 1, presented one at a time and in randomised order in the centre of the computer screen). Each word was presented four times, and on each occasion, the words were displayed on the screen until the correct key was pressed or 2 s had elapsed. The next word was presented 2 s after the previous word. The selection of emotional words most relevant to the content shown in our three videos was determined in a prior pilot study in which 16 words (8 positive and 8 negative) were identified from a cohort of 50 words as being ranked most closely to the emotions elicited in the videos and categorised consistently as of positive or negative valence.

On each trial, an emotional word appeared briefly on the computer screen and subjects were instructed to categorise them according to their emotional valence by pressing the “I” key on the keyboard if the word was positive in nature and the “E” key if the word was negative (key mapping was counterbalanced between the subjects). A reminder of which key corresponded to which emotional valence “Positive” or “Negative” remained on the computer screen in the top left and right corners throughout the two phases. Subjects were asked to respond as quickly and as accurately as possible. 

The baseline trials and the experimental trials were identical, except that a video was played in the background during the experimental trials. The baseline phase of the Impulse test served both as a means of training the subjects to respond within a very short timeframe (to clear contamination from conscious brain processes) and also to familiarise the participants with the task. Responses that were deemed too slow to be classified as pre-cognitive were followed by a brief warning tone and the visual warning “too slow”. Following successful completion of the training phase, subjects were informed via instructions on the computer that the experimental phase was about to begin. The design was similar to the practice phase, however, during this phase, the words appeared superimposed on the dynamic footage (the three videos are described in the Materials section). Respondents were instructed to continue classifying the words as positive or negative in terms of emotional valence and to press the corresponding keys on the computer keyboard.

Our previous research has shown that the speed and accuracy of classification of these words reflect the feelings that a participant has towards the content of the movie or television clip [34]. By comparing reaction times to classify positive and negative emotional words during the training (baseline) and experimental phase, it is possible to infer the nature of the internal feelings elicited in the viewer by the video content every 2 s. Specifically, we have found that positive emotions elicited by the footage shown, speeds responses to positive words and slows them to negative ones. The reverse holds true for aspects of the footage that elicits negative emotions—responses to negative words are sped up and responses to positive words are slowed. In both cases, RTs were computed against the responses recorded during the baseline and training phase, for each participant. To understand this approach in the context of semantic priming studies, in the current study, the video content acts as the “priming” stimulus, with the emotional words being the targets.

#### 3.1.4. Analysis

The physiological analysis focused on the emotional peaks detected whilst watching each video. We hypothesized that during each emotionally provocative video, there were likely to be fluctuations in arousal, as determined by changes in electro-dermal response, breathing rate and heart rate. We also hypothesized that these physiological indices would be accompanied by changes in implicit psychological emotional responses detected using the Impulse test. 

##### Physiological Responses

**Figure 1:**
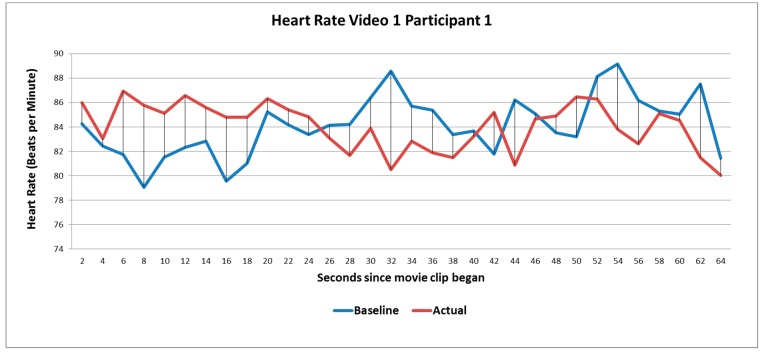
This graph shows the heart rate of one representative participant when they were carrying out the baseline test (blue line) and the test with the movie clip in the background (red line). The resulting data computed for this participant is the difference between the blue and red heart rate values every two seconds. When the value of a point on the red line is larger than the value of the corresponding point on the blue line (e.g., at t = 6), it shows that the participant’s heart rate increased as a result of watching this part of the movie clip. Conversely, at t = 28, the participant’s heart rate shows a decrease. These values were computed for each participant and then averaged and subjected to statistical analysis.

For the physiological data, the maximum values for each physiological measure (heart rate, breathing rate and skin conductance) were first computed by extracting the peak response recorded every 2 s during both the training and experimental phase of the Impulse test and the results from all individuals averaged and tested for statistical significance using paired T-tests at each time point (see Figure 1). 

##### Impulse Test

Reaction time data obtained during the training and experimental phases of the Impulse test were first subjected to pre-processing, including removal of outliers so that responses that were impossibly quick (<250 ms) and those that were so slow as to invoke conscious processing (>1200 ms), were removed. The data were then analysed following the method outlined by Fazio and Olson [36]. For all trials of each word presented in each video, a facilitation index (FI) was computed. For all congruent responses (e.g., classifying “delightful” as “Positive”) obtained for each word across all trials, the FI was computed by subtracting the reaction times during the experimental phase from those obtained during the baseline phase. For incongruent responses to each word (e.g., classifying the word “lonely” as “Negative”), the FI calculation was reversed such that reaction times obtained during the baseline phase were subtracted from the reaction times obtained during the experimental phase. Thus, an FI greater than zero implies a response that is congruent with the emotion word set and a FI less than zero implies a response incongruent (or opposite) with the word set. This approach allowed us to take into account both the congruency (or subjective accuracy) of responses as well as the reaction times. The dependent variable was, for each moment of each video (every two seconds), the percentage of participants whose FI indicated that the footage at each time-point was either congruent or incongruent with a positive emotion or negative emotion. The averaged data were then tested for each condition for statistical significance using the binomial test that computes the probability of obtaining a specific count in one direction (e.g., a positive emotional response) against the total number of observations (the number of positive and negative responses).

#### 3.1.5. Results

##### Physiological Measures

Condition 1: (Everyday pleasures) While viewing a video depicting everyday pleasures, breathing rate dropped from 16.3 cycles to 15.4 cycles per minute); heart rate remained stable at 76.1BPM in both cases; and a non-significant increase in electrodermal response from 0.171 to 0.252 was recorded.

Condition 2: (Providing good customer service) was associated with an average increase in heart rate from 76.0 BPM during the baseline phase to 87.4 BPM while the video was shown in the background (*p* < 0.01). Breathing rate decreased from 16.7 cycles per minute during the baseline to 10.2 cycles per minute while viewing the video, and a significant increase in electrodermal response from 0.114 to 0.335 (*p* < 0.001) was recorded.

Condition 3: (Receiving good customer service). A statistical comparison of physiological measures revealed that viewing footage of others receiving excellent customer service resulted in a significant increase in electrodermal response from 0.164 to 0.308 (*p* < 0.01) and a significant decrease in heart rate from 71.4 to 80.6 BPM (*p* < 0.05). There were no significant differences in breathing rate for condition 3 (17.2 to 16.8).

##### Impulse Test

The control condition (viewing everyday pleasures) elicited an FI of −3.15, showing that that there was a slightly shorter mean response latency to negative attributes than to positive attributes. However, this FI did not differ from zero (*p* > 0.05). Viewing footage of individuals receiving excellent customer service elicited an FI of +36.9, which reveals a significant increase in response latency to positive attributes (*p* < 0.001). Viewing footage of individuals providing excellent customer service yielded the largest increase in FI of +53.8 (*p* < 0.001). Paired t-tests revealed that providing excellent customer service elicited a greater association with positive emotions than either receiving excellent service (*p* < 0.05) or viewing everyday pleasures (*p* < 0.001). 

#### 3.1.6. Discussion and Conclusion

We believe that this is the first demonstration that viewing instances of positive customer service interactions from the perspective of both the recipient and the service provider has a positive impact on physiology and emotional well-being. Specifically, viewing footage of people delivering or receiving excellent customer service resulted in a significant increase in arousal levels, as evidenced by the increase in galvanic skin response and a significant decrease in heart rate (compared to viewing scenarios of everyday pleasures), indicating that positive customer service interactions can have a stress-reducing and calming impact on the service provider and surrounding viewers. 

The results of the Impulse reaction time study showed that participants were faster at correctly classify positive word targets than negative ones when viewing footage of people providing good service compared to receiving it, or while viewing footage of every day pleasures. This is an intriguing finding as we would have hypothesized that people would adopt a self-interested stance and would instinctively attach greater positive valence to receiving good service than watching examples of people providing good service. Receiving good service was perceived with the same level of positive emotional engagement as viewing every day pleasures, highlighting the growing significance of customer service in people’s lives today.

The results of Study 1 raised further questions relating to the generalizability of these findings across different countries, age groups and gender. Therefore, in the next study, we sought to extend these findings by investigating the implicit emotional feelings associated with both positive, as well as negative, customer service interactions in a larger population using a web-based implicit affective priming task designed to uncover the strength of emotional association that people hold about positive and negative customer service interactions.

### 3.2. Study 2: Online Study

#### 3.2.1. Participants

Participants (N = 1200) from three countries (UK, Canada and Australia; N = 400 from each country, 50% males) were recruited through a research participation recruitment company (Research Now) and were given small incentives to complete the tests. All the participants had normal to corrected vision, were native English speakers between 18 and 60 years and completed an online consent form prior to participation (the sample size is adequate for the chosen experimental design, since the study is a four (providing excellent or poor customer service vs. receiving excellent or poor customer service) by two (excellent service vs. poor service) design and is similar to other studies of comparable nature, e.g., [35,40].

#### 3.2.2. Materials

The web-based survey included three components: (i) demographic questions to confirm age, gender, handedness and previous employment in a service industry, (ii) a consent form, and (iii) an implicit affective priming task. The survey was programmed in Javascript so that as soon as participants entered the survey, the test would automatically and immediately be downloaded onto their pc/laptop so that reaction times could be captured using the internal timing devices on the pc/laptop, which are far more sensitive than if running a program of this nature across the internet. On completion of the survey, the individual datasets were then uploaded back onto the server for analysis and without being apparent to the participant.

The affective priming task consisted of a series of emotional word primes (Table 2) and target statements (Table 3). The emotional word primes were selected following an explicit pilot test in 150 people (50 from each country) during which respondents were asked to classify attributes (from a set of 60; including those used in Study 1) into those most likely to be experienced in the context of extremely pleasurable experiences, peace of mind experiences, everyday experiences, and negative experiences. Of these, 35 were consistently classified and used as primes in the implicit test. The brief statements used as targets (e.g., “being helpful”, “feeling relieved”) were generated in consultation with service industry consultants and refer to the behaviours that were most often experienced in the context with excellent or poor service scenarios (Table 3).

#### 3.2.3. Protocol

On entering the survey, respondents were asked to confirm their age, gender and handedness. They were also asked if there were currently employed, and/or did voluntary work and whether their current or any past employment involved “providing service of some form to service users, such as clients, customers or patients”. If they answered “no” to the last question, they were thanked for participating but informed that they were not eligible for the study. 

On completing the inclusion criteria questions and subsequent consent form, participants were then asked to classify each of the 35 emotion words (pre-selected for inclusion in the implicit test) as extremely pleasurable experiences, peace of mind experiences, mundane experiences and negative experiences.

Participants were then instructed that they would be asked to perform a reaction time task that would measure how quickly and accurately they could classify a series of short phrases (see Table 3) that would be presented in the centre of the computer screen. There were two tasks designed to identify emotions implicitly associated with *providing* excellent or poor customer service (Test A) or *receiving* excellent or poor customer service (Test B). Participants were randomly assigned to one of the two tasks.

Before the experimental trials, participants were given 24 practice trials during which they were asked to discriminate whether short phrases which were either positive or negative (e.g., “being helpful”, “being impolite” in the case of test A—*providing* excellent or poor service) and (e.g., “feeling special”, “feeling neglected” in the case of test B—*receiving* excellent or poor customer service) were synonymous with either “excellent service” or “poor service” and to press the “E” or “I” key on the computer keyboard corresponding to each option. The practice trials served as a learning phase during which respondents were able to learn the association between each target and the correct key press so that they would not need to focus on which key to press during the main test. 

The keys were allocated to “excellent service” or “poor service” and were counterbalanced for each participant, and once assigned, remained so for the duration of the task. If a response was incorrect, the error message “Try again!” appeared near the lower part of the screen; if two keys were pushed at the same time, the message “Please press only one key at a time” was displayed; if no key was pushed within two seconds, the cue “Warning: Please press E or I” appeared. The next trial proceeded after a 1500 ms inter-trial interval. Participants were instructed to respond as quickly as possible but to avoid making a mistake. 

Following practice trials, participants were told that the main trials were about to begin and would be very similar as the practice phase but this time a word or “prime” was presented for 500 ms, immediately before the short phrase targets. Each prime was presented four times in total, twice before a phrase associated with “excellent service” and twice before a phrase associated with “poor service” to ensure a sufficient number of trials of each type. Prior testing has shown that with an N = 1200, this number of trials is sufficient to be able to detect a statistical difference if it exists. The task was identical to that conducted during the practice trials, which was to discriminate whether the targets (e.g., “being” or “feeling” a positive or negative emotion) that appeared immediately after the primes (see Table 2) were associated with “excellent service” or “poor service” and to respond as quickly and as accurately as possible by pressing the key corresponding to each option.

#### 3.2.4. Analysis

Data were first subjected to analysis to remove outliers, including response times that were impossibly fast (<250ms) or those that occurred after the permitted time window. Reaction times were then computed for each word attribute and for each participant. A difference score was computed being the mean reaction time when the prime was presented before ‘poor service’ minus the mean reaction time when the prime was presented before ‘excellent service’. This was also done separately for Tests A (Providing) and B (Receiving). Positive difference scores indicated that a prime was more strongly associated with “excellent service” than with “poor service”. Negative difference scores indicated the reverse. Difference scores greater than zero were recoded as +1 and difference scores less than zero were recoded as −1 (scores at zero were not included in the subsequent analyses). For each attribute, we then computed the percentage number of 1 s, this value would reflect the percentage of participants who more strongly associated the prime with excellent service than with poor service.

#### 3.2.5. Results

The results focus on the comparison of emotional attributes that were both significantly associated with providing versus receiving positive and negative customer service, as well as the overall number of positive and negative emotions attributed to each condition. Here, we first report the results for the entire group (averaged across all countries).

##### Providing Excellent Customer Service

The analysis of reaction times recorded when participants classified the targets subsequent to emotional primes in Test A (Providing customer service) found that (collapsed across all countries) providing excellent service was associated with fastest responses to feeling “calm” and “proud” (*p* < 0.001). Other emotional word attributes that were found to be strongly associated with providing excellent service included feeling “fair”, “engaged”, “loved” and “pleased”, “nice”, “okay” and “ecstatic” (*p* < 0.05). A total of nine positive emotion words were found to be implicitly associated with providing excellent customer service. 

##### Receiving Excellent Customer Service

Receiving excellent service (Test B) was found to be associated with faster responses when preceded by the primes “energised”, “happy” and “proud” (*p* < 0.001). Other emotions that were also strongly associated with receiving excellent customer service were “calm”, “satisfactory”, “nice”, “fair” and “okay” (*p* < 0.05), attributes that were previously categorised as being experienced when engaging in everyday pleasures, such as meeting friends.

**Figure 2:**
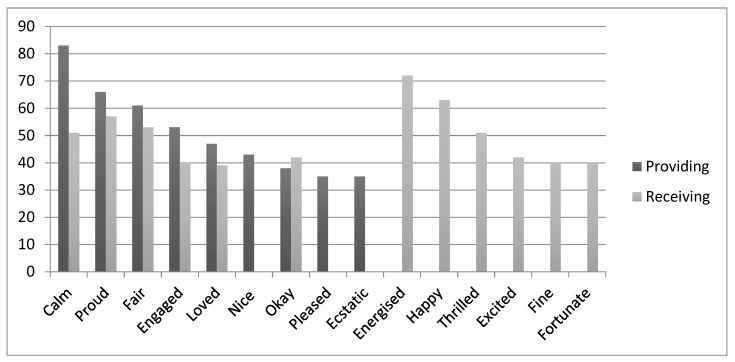
Emotional attributes associated with providing versus receiving excellent customer service (Y-axis shows the percentage of people significantly associating primes with the receipt and provision of excellent service).

A comparison of significant associations across the two tests (see also Figure 2) revealed that only providing excellent service was associated with “pleased” and “ecstatic”, whereas receiving excellent service elicited significant associations with the attributes “energised”, “happy”, “thrilled”, “excited”, “fine” and “fortunate”. 

##### Providing Poor Customer Service

Providing poor customer service was significantly associated with the emotional attributes, “lonely”, “nervous”, “sad” and “annoyed”. 

##### Receiving Poor Customer Service

**Figure 3:**
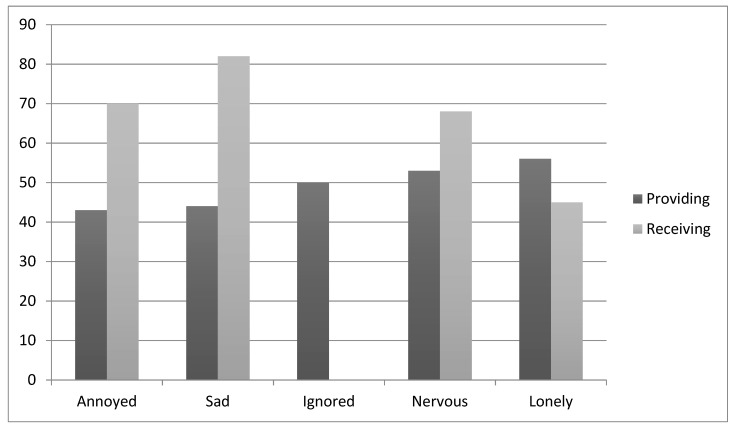
Emotional attributes associated with providing versus receiving poor customer service (Y-axis shows the percentage of people significantly associating primes with the receipt and provision of poor service).

Receiving poor customer service was significantly associated with these same four emotional attributes and additionally, with feeling “ignored”. Receiving poor service was more strongly associated with the attributes “sad” and “annoyed” than was providing poor service (see also Figure 3).

##### Gender Differences

The statistical comparison of males and females collapsed across all countries found that while females associated more positive attributes with receiving excellent customer service, males associated more positive attributes with providing excellent customer service (both *ps* < 0.01). Specifically, females associated receiving excellent service with feeling “happy”, “energised”, “over-joyed”, “proud”, “thrilled”, “exhilarated”, “loved”, “nice”, “expected”, and “fair”. Receiving poor service was more significantly associated with feeling “nervous”, “sad” and “lonely”. By comparison, providing excellent service was associated with feeling “proud”, “calm”, “pleased”, “fair”, and “engaged”, whereas providing poor service was associated with feel “sad”, “annoyed” and “lonely”. 

Males were faster to associate the provision of positive customer service with feeling “energised”, “calm”, “engaged”, “proud”, “thrilled” and “nice”. Providing poor service made them feel “nervous”. Receiving excellent customer service was associated with feeling “satisfactory”, “calm”, “okay”, “satisfied”, “ecstatic”, ”engaged”, “relief” and “nice”. Receiving poor customer service was associated with feeling “annoyed”, “regular” and “lonely”.

##### Age Differences

Respondents aged between 18 and 35 years associated more positive attributes with receiving than providing excellent customer service (*p* < 0.001), including “OK, fair, confident, nice, satisfactory, engaged, energised, thrilled, calm, ecstatic, exhilarated, content, happy, pleasant”. Receiving poor service was associated with feeling “annoyed”, “sad” and “lonely”. Providing excellent service was associated with feeling “calm”, “engaged” and “proud” and “OK”; providing poor service made them feel “sad”, “ignored” and “nervous”.

In stark contrast, respondents in the older age group (36+) associated more positive attributes with providing rather than receiving excellent customer service (*p* < 0.001). Specifically, providing excellent service was more closely associated with feeling “proud” and “calm”, “excited”, “pleased”, “nice” and “fair”. Providing poor service made them feel more “annoyed” and “sad”. Receiving excellent service made the older group feel “proud” and “calm”, poor service interactions made them feel “nervous”, “lonely”, “ignored”, “regular” and “sad”.

##### Cross-Cultural Differences

There were also a number of interesting cross-cultural differences in terms of the emotions most closely associated with providing and receiving good and poor customer service. 

##### United Kingdom (least Impacted by Customer Service- Expectations much Lower)

Comparison of the statistical effect sizes between countries revealed that while respondents in the United Kingdom showed a positive association between giving or receiving amazing service, the effect was lower than that recorded for Canada and Australia. 

##### Canada (Focused on “Providing”)

It was noteworthy that Canadians felt more “thrilled”, “content” and “pleased” when providing rather than receiving excellent service. Receiving, rather than giving amazing service was, on the other hand, more associated with a positive association with the emotions, “exhilarated”, “energised”, “happy”, “loved”, “relieved”, “pleasant” and “fine” (*p* < 0.05). 

##### Australia (Receiving is More Emotionally Important than Giving)

Australians were found to associate the provision of amazing service with a sense of “calm” (*p* < 0.05), compared to receiving the same level of service. Australians were statistically more likely to feel “fortunate”, “thrilled”, “happy” and “appreciated” when receiving excellent service compared to when they were providing it.

#### 3.2.6. Conclusions

In study 2, we demonstrated the implicit association of positive and negative feelings with proving and receiving good customer service across a large general populace. We also show the generalizability of our results across three cultures, ages and genders. Specifically, we demonstrated that, (1) providing and receiving excellent customer service was strongly associated with certain emotions (feeling “calm”, “proud”, “fair”, “engaged”, “loved”, “pleased”, “nice”, “okay”, “ecstatic”, “energised”, “happy” and “satisfactory”), and (2), providing and receiving poor customer service was strongly associated with certain emotions (feeling “lonely”, “nervous”, “sad”, “annoyed” and “ignored”), (3) females associated providing and receiving excellent customer service with certain emotions (“happy”, “energised”, “over-joyed”, “proud”, “thrilled”, “exhilarated”, “loved”, “nice”, “expected”, “fair”, “calm”, “pleased” and “engaged”), (4) females associated providing and receiving poor customer service with the emotions “nervous”, “sad”, “lonely” and “annoyed”, (5) males associated providing and receiving excellent customer service with the emotions “energised”, “calm”, “engaged”, “proud”, “thrilled”, “nice”, “satisfactory”, “okay”, “satisfied”, “ecstatic” and “relief”, (6) males associated providing and receiving poor customer service with the emotions (“nervous”, “annoyed”, “regular” and “lonely”). We also found that younger respondents associated more positive attributes with receiving, rather than providing, excellent customer service, whereas older respondents associated more positive attributes with providing rather than receiving excellent customer service. Among cross-cultural differences, we found that in (1), UK respondents showed a weak association between giving or receiving an amazing service and their expectations were lower (compared to Canada and Australia), (2) Canadian respondents showed a stronger association for providing rather than receiving excellent service and (3), Australian respondents showed a stronger association for receiving rather than providing excellent service).

## 4. General Discussion

In the current study, we exploited two implicit reaction time tasks. The first, the recently developed Impulse test, is a novel implicit reaction time paradigm that measures the moment-to-moment shifts in emotions when, for example, people are viewing dynamic videos or footages [34]. The second is a task based on affective priming, a very well established implicit paradigm that was developed out of cognitive psychology in the 1980s [41,42,43] and has been recently adapted for use in commercial neuromarketing studies [35]. Both implicit tasks are ideal for capturing the complex, often subconscious, emotions associated with receiving and providing customer service of varying quality in order to understand the subtle impact of these customer–staff interactions on emotional well-being. Major advantages of using these methods are that they are indirect and are not as susceptible to the response biases associated with explicit responses (e.g., self-reported measures) and that they can reveal the moment-to-moment scores during a video clip, rather than a post test score.

Our results show that people do not only find *receiving* excellent customer service as pleasurable but *providing* excellent service is equally satisfying. We corroborate these results using both physiological measures (study 1) and an implicit reaction time paradigm (study 2). We also provide evidence that both giving and receiving excellent service can actually reduce stress and anxiety levels amongst both consumers and service providers and have a positive impact on their wellbeing. These results were shown to hold true across three countries, demonstrating that giving and receiving excellent customer service can induce a sense of pride, calmness and of being loved.

Our data additionally revealed some age and gender differences. Specifically, our results reveal that younger individuals (18–35 years) exhibit more positive emotions when receiving than giving good customer service, whilst the opposite was the case for older participants. In thinking about being served, relatively more focus is placed on oneself (vs. others); in thinking about providing service, relatively more focus is placed on others (vs. oneself). Therefore, our results suggest that younger people tend to focus more on themselves (vs. others), whereas older individuals focus more on others (vs. themselves). This pattern of findings is consistent with Freund Blanchard-Fields’ [44] observation that older adults are more altruistic (i.e., focusing on the needs of others rather than on themselves) than younger adults, and tend to behave in ways that benefit others rather than themselves (e.g., donating money to a good cause rather than keeping it for themselves). By contrast, younger adults tend to focus on maximizing their personal gains over the interests of other people. Collectively, these findings add to the existing knowledge about customer service by underscoring the importance of age differences when it comes to customers and service providers. Future research may test the altruism explanation for the observed effects due to age differences. 

Analysis of gender differences revealed that females tend to prefer receiving (vs. providing) excellent service, whereas the reverse is true for males. At first glance, this finding appears somewhat contradictory to past research that suggested that women are generally communal, warm, and nurturing, whereas men tend to be more competitive and goal-oriented [45,46]. However, we interpret this finding in the light of other research which showed that men and women place a different emphasis on different aspects of service. While men are usually more concerned about the core aspect of the service (e.g., the haircut received at a hair salon), women generally pay more attention to the relational aspects of service (e.g., how well one gets along with the hairstylist) [47]. It is also likely that the core (relational) aspects of service are more salient when thinking about giving (receiving) excellent customer service because the focus is on helping the recipient resolve their problem (core aspect); in thinking about receiving service, it is easier to think about how one would feel about being served (relational aspect). Applying this to the gender differences that we found, it is possible that men preferred giving (vs. receiving) excellent service because it is more closely aligned with their goal-oriented tendency. Women, on the other hand, preferred receiving (vs. giving) excellent service as they were drawn towards its more highly salient relational aspects of service as they imagine themselves being served. Future research may follow this lead to explicitly examine the underlying processes driving the results that we observed through the implicit tests. 

### 4.1. Theoretical and Methodological Contributions

To the best of our knowledge, this research is the first to employ two implicit tests, targeting both individual and group level responses, in order to yield a comprehensive view of the payoffs of good customer service. This current research also contributes to retailing research, which tends to focus on explicit data, by adding the implicit angle to understand how customer service impacts individuals at a subconscious level.

### 4.2. Implications for Managers and Organizations

Past research on customer service is heavily focused on understanding how customer service affects the customer and how satisfied customers in turn reward organizations with increased sales, patronage, and higher profits. Service personnel, who often shoulder the “burden” of delivering customer service that yields benefits to customers and organizations, appear to gain the least from the exchange. Our current research augments this stream of literature by focusing on what customer service means to service providers. Managerially, the observation that older people exhibit a preference for providing good customer service suggests that companies might wish to consider employing more mature individuals on the front line (albeit with due consideration of the physical requirements related to standing in-stores for long hours) because they may be more naturally inclined to servicing the needs of others. In addition, we believe that our results gain credibility from the fact that for the implicit reaction time test, the primes chosen (e.g., pleasant experiences) were selected by real consumers and the targets (e.g., “being helpful”) were chosen in consultation with service industry consultants. 

Our study found that service providers also benefit from delivering good customer service in the form of enhanced emotional well-being and inoculation against negative, damaging emotions. To some extent, understanding that delivering good customer service is emotionally lifting to the service providers helps to resolve the pressure of having to engage in acting to please customers. In the emotional labour literature, researchers identified two levels of acting—surface (where the employee displays false emotions that s/he does not feel, only to please customers) and deep (where the employee feels the emotions that she/he displays to customers)—that service personnel use when dealing with customers. However, both surface and deep acting have potential problems. Surface acting is often perceived as fake and distancing to customers; on the other hand, deep acting places considerable emotional strain on the service provider. Based on our results, service providers can be coached to focus on understanding how delivering good service makes them feel and the subsequent emotional payoffs they can gain from it. This may help to reduce employee burn-out and turnover whilst maintaining happy customers and a healthy bottom-line. Therefore, training employees to focus on how good customer service benefits themselves creates a positive feedback loop that benefits customers, service providers, and organizations alike. 

## Figures and Tables

**Table 1:** Emotional words used during the Impulse test.

Positive Valence Words	Negative Valence Words
Excited	Weepy
Over-joyed	Stressed
Delighted	Sad
Contented	Heartbroken
Pleased	Lonely
Ecstatic	Ignored
Peaceful	Fed up

**Table 2:** Emotional prime words used in the affective priming task.

Emotional Prime Words						
Excited	Confident	Content	Appreciated	Regular	Okay	Sad
Ecstatic	Fortunate	Comforted	Peaceful	Satisfactory	Fine	Lonely
Over-joyed	Engaged	Pleased	Relief	Pleasant	Normal	Ignored
Exhilarated	Proud	Happy	Calm	Nice	Expected	Annoyed
Energised	Thrilled	Loved	Satisfied	Fair	Usual	Nervous

**Table 3:** Emotional words used to create brief statements used in Test A (Providing) and Test B (Receiving). All target words using in Test A were presented prefixed with the word “being” (e.g., “being helpful”, “being friendly”), whereas those used for Test B were pre-fixed with the word “feeling” (e.g., “feeling relieved”, “feeling neglected”).

Providing Service Targets “Being”		Receiving Service Targets “Feeling”	
Positive	Negative	Positive	Negative
Helpful	Impolite	Relieved	Neglected
Friendly	Difficult	Special	Insecure
Sensitive	Confusing	Understood	Misconstrued
Excellent	Lazy	Encouraged	Ignored
Understanding	Thoughtless	Unique	Angry
Supportive	Rude	Respected	Insulted
Considerate	Cold	Wowed	Underwhelmed
Meaningful	Useless	Protected	Frustrated
